# The Impact of Inaccurate Internet Health Information in a Secondary School Learning Environment

**DOI:** 10.2196/jmir.986

**Published:** 2008-06-30

**Authors:** Philip Kortum, Christine Edwards, Rebecca Richards-Kortum

**Affiliations:** ^2^University of Texas Health Science Center School of MedicineSan AntonioTXUSA; ^1^Department of Psychology, Rice UniversityHoustonTXUSA

**Keywords:** Health education, health informatics, popular medicine, vaccines

## Abstract

**Background:**

Patients in the United States commonly use the Internet to acquire health information. While a significant amount of health-related information is available on the Internet, the accuracy of this information is highly variable.

**Objectives:**

The objective of the study was to determine how effectively students can assess the accuracy of Internet-based material when gathering information on a controversial medical topic using simple keyword searches.

**Methods:**

A group of 34 students from the science magnet high school in Houston, Texas searched for the terms “vaccine safety” and “vaccine danger” using Google and then answered questions regarding the accuracy of the health information on the returned sites. The students were also asked to describe the lessons they learned in the exercise and to answer questions regarding the strength of evidence for seven statements regarding vaccinations. Because of the surprising revelation that the majority of students left the exercise with inaccurate information concerning the safety and efficacy of vaccines, these same students participated in a follow-up study in which a fact-based vaccine video was shown, after which the assessment of student knowledge was repeated.

**Results:**

Of the 34 participants, 20 (59%) thought that the Internet sites were accurate on the whole, even though over half of the links (22 out of 40, 55%) that the students viewed were, in fact, inaccurate on the whole. A high percentage of the students left the first exercise with significant misconceptions about vaccines; 18 of the 34 participants (53%) reported inaccurate statements about vaccines in the lessons they learned. Of the 41 verifiable facts about vaccines that were reported by participants in their lessons-learned statement, 24 of those facts (59%) were incorrect. Following presentation of the film, the majority of students left the exercise with correct information about vaccines, based on their lessons-learned statement. In this case, 29 of the 31 participants (94%) reported accurate information about vaccines. Of the 49 verifiable facts about vaccines that were reported by participants, only 2 (4%) were incorrect. Students had higher correct scores in the “strength of evidence” exercise following exposure to the video as well.

**Conclusions:**

Allowing students to use the Internet to gain information about medical topics should be approached with care since students may take away predominantly incorrect information. It is important to follow up conflicting information with a solid, unambiguous message that communicates those lessons that the instructor deems most important. This final message should be fact based but may need to contain an anecdotal component to counter the strong emotional message that is often delivered by inaccurate Internet sites.

## Introduction

Use of the Internet for the acquisition of health information in the United States is widespread and growing [1]. Whether looking for information about disease symptoms or treatment options, health consumers see the Internet as an important tool for gathering health information. One benefit of the Internet is its potential to provide current and timely sources of health information in ways that traditional print resources cannot. This is extremely important in the health domain since information may change rapidly and currency of information can be very important. The Internet also provides health consumers with a wide range of no-cost material that is easy to access. Printed materials carry acquisition costs in terms of both time and money and may be difficult to obtain for those who are not health professionals, leading consumers to use the Internet [2]. Clearly, the advantages of obtaining health information via the Internet make it an attractive source for the average health consumer.

Unfortunately, these advantages do not come without some cost. Because information is available quickly, it can be transient in nature, and excellent sources found today may not be available for use again in the future. While there are many sources on the Internet, the accuracy of all these sources may not be the same. Worse, the accuracy of specific content is difficult to ascertain quickly and easily. This means that consumers of Internet health information must have good scientific literacy to be able to sift thorough copious amounts of information and make an informed choice about which to keep and which to discard. Because of this abundance of information of unknown accuracy, consumers of this information typically employ a number of different strategies to aid them in their efforts [3].

For many health issues, there is widespread agreement in the medical community about the proper course of action (eg, how to treat a minor cut). In other situations, there may be different opinions about the correct course of treatment (eg, angioplasty vs coronary artery bypass) based on the specific medical facts of a particular case or how an individual patient presents. These kinds of differences are understandable and are inherent in the practice of medicine. Other topics, however, while enjoying widespread agreement in the mainstream medical community, are still the source of significant controversy due to the efforts of some outspoken groups and individuals. The Internet provides these groups a strong voice by allowing them to share their views in a manner similar to sources of medical information that are generally accepted as authoritative. On the Internet, medical information from trusted sources like the American Medical Association or the National Institutes of Health must compete with information from groups and individuals who may not be trained in the field or who may interpret data in unscientific ways that support their particular viewpoints. Even the best Internet search engines do not return results in the order of authenticity or trustworthiness of the source, and the brief descriptions that are included in search results do not provide sufficient information for a consumer to make a well-informed assessment of the accuracy or reliability of the site [4]. This makes finding and evaluating this type of health information on the Internet particularly difficult [5].

Information about vaccines falls into the category of medical topics that have a high degree of controversy between the mainstream medical establishment and groups who disagree with the generally accepted lines of thought. For the most part, vaccines are considered to be one of the most important medical advances, eradicating or significantly reducing mortality from a host of now-preventable diseases. When administered according to well-established protocols, vaccines are considered both safe and effective. Their impact in developing countries is especially important since many diseases that are relatively rare in the developed world still claim large numbers of lives [6]. Despite the scientific evidence that illustrates the benefits of vaccines, there are a number of groups who espouse the contrary view that vaccines are actually harmful and may cause, rather than prevent, disease [7], and the number of parents in the United States who choose not to vaccinate their children continues to grow [8].

As part of an effort to teach students to gather and evaluate health information, we developed an exercise that had them seek out information about vaccines using the Internet as their primary data source. The goal of this research was to determine how effectively students could assess the accuracy of Internet-based material when gathering information on a controversial medical topic using simple keyword searches. Based on this teaching experience, we make suggestions about the design of instruction material for similar exercises.

## Methods

### Methods for the Search Exercise

A group of 34 juniors and seniors from the science magnet high school in Houston, Texas (Milby Science Institute) were recruited to participate in the study. In the United States, magnet schools are schools that draw academically talented students from a wide geographic area to allow them to focus on a specific concentration of study, like music, science, or mathematics. Participants were volunteers from an advanced science class at Milby. There were 17 males and 17 females. Although ethnicity data were not collected from the participants, Milby is comprised of 94% Hispanic, 4% African American, 1% Asian, and 1% White students. Although Milby is predominantly Hispanic, its minority enrollment is similar to that of the Houston Independent School District as a whole. The Houston Independent School District is the seventh largest public school system in the nation, and with almost 200,000 students, is the largest in Texas. Its student body is 60% Hispanic, 28% African American, 8% White, and 3% Asian. At Milby, 79% of the students are classified as economically disadvantaged, as defined by the federal guidelines for participation in the free/reduced-price lunch program, and this percentage is identical to the Houston Independent School District as a whole. This demographic is representative of that found in most large urban educational environments.

**Table 1:** Accuracy judgments of sites that were returned by the Google search for “vaccine danger”

Search Result Position	Site URL	Google Title Description	Accurate?
1	www.know-vaccines.org/parent.html (http://www.webcitation.org/5YKI9b32b)	Know Vaccines – Contact Information	No
2	www.nccn.net/~wwithin/vaccine.htm (http://www.webcitation.org/5YKIKHZTF)	Vaccination Information & Choice Network - Vaccine/Vaccination	No
3	www.cbsnews.com/stories/2003/08/21/eveningnews/main569522.shtml (http://www.webcitation.org/5YKIW0Ycm)	Military Mute On Vaccine Danger?	No
4	www.909shot.com/History/Newsletters/nlr1296.htm (http://www.webcitation.org/5YKIcRpCq)	The Vaccine Reaction	No
5	www.shirleys-wellness-cafe.com/vaccines.htm http://www.webcitation.org/5YKIiwNgh)	Vaccines Warning: Are they really safe and effective?	No
6	www.shirleys-wellness-cafe.com/petvacc.htm (http://www.webcitation.org/5YKIo2L58)	Danger of Pet Vaccination - Vaccinosis - adverse reaction to ...	No
7	www.thinktwice.com/multiple.htm (http://www.webcitation.org/5YKIuVzbs)	ThinkTwice Global Vaccine Institute: Multiple Vaccines. Danger!	No
8	www.veteransforpeace.org/Military_mute_on_082103.htm (WebCite not available)	Military Mute on Vaccine Danger?	No
9	www.mercola.com/forms/vaccine_teleconference.htm (http://www.webcitation.org/5YKJDuxJl)	The Danger of Vaccines, and How You Can Legally Avoid Them	No
10	educateyourself.org/cn/infantimmunizationweek14apr05.shtml (http://www.webcitation.org/5YKJIvWaw)	National Infant Immunization week	No
11	www.shirleyswellnessnews.com/n/n11-02.htm (http://www.webcitation.org/5YKJNehtW)	Shirley’s Wellness Café Newsletter	No
12	Inquirer.gn.apc.org/rubella2.html (http://www.webcitation.org/5YKJa9ElJ)	Gambling with Rubella Vaccine	No
13	www.909shot.com/Articles/gnspriva.htm (http://www.webcitation.org/5YKJyv8RA)	Vaccination Nation	No
14	www.cbsnews.com/stories/2004/03/01/eveningnews/main603284.shtml (http://www.webcitation.org/5YKK4FTBM)	Military Vaccine Flattens GI, 17	No
15	Bmj.bmjjournals.com/cgi/eletters/316/7129/446 (http://www.webcitation.org/5YKK9vPQH)	Bmj.com Rapid Response for Masters and Beyreuther, 316(7129)446-448	Yes
16	www.eczemavoice.com/forum/messages/270/441.html?1041724527 (http://www.webcitation.org/5YKkX1cYE)	Eczema Voice	No
17	Society.guardian.co.uk/publichealth/story/0,,588304,00.html (WebCite not available)	SocietyGuardian.co.uk | Society | New claims of vaccine danger	Yes
18	www.geocities.com/heartland/8148/vac.html (http://www.webcitation.org/5YKkhJnNU)	Be informed about vaccines	No
19	http://www.advancedhealthplan.com/mothersday.html (http://www.webcitation.org/5YKl0YaP7)	Mothers day Proclamation Original by Julia Ward Howe.	No
20	www.whale.to/v/tebb/ap7.html (http://www.webcitation.org/5YKl3ani5)	Compulsory Vaccination in Bombay	No

**Table 2:** Accuracy judgments of sites that were returned by the Google search for “vaccine safety”

Search Result Position	Site URL	Google Title Description	Accurate?
1	www.vaccinesafety.edu/ (http://www.webcitation.org/5YKlm91Os)	Institute for Vaccine Safety (IVS)	Yes
2	www.vaccinesafety.edu/thi-table.htm (http://www.webcitation.org/5YKonwPOM)	Institute for Vaccine Safety – Thimerosal Table	Yes
3	Vaccines.net/newpage114.htm (http://www.webcitation.org/5YKlyCebp)	Vaccine Safety	No
4	www.cdc.gov/nip/vacsafe/ (http://www.webcitation.org/5YKndg6d3)	NIP: Vacsafe/Overview (main Page)	Yes
5	www.cdc.gov/nip/menus/vacc_safety.htm (http://www.webcitation.org/5YKoLsNzP)	NIP: Menus/Vaccine Safety	Yes
6	www.909shot.com/ (http://www.webcitation.org/5YKmwGu3k)	National Vaccine Information Center	No
7	www.immunize.org/safety/ (http://www.webcitation.org/5YKmzAt57)	Vaccine safety information form IAC	Yes
8	www.who.int/immunization_safety/en/ (http://www.webcitation.org/5YKn2I3N0)	WHO | Immunization safety	Yes
9	www.fda.gov/Fdac/features/095_vacc.html (http://www.webcitation.org/5YKn6l54C)	How FDA works to insure vaccine safety	Yes
10	www.vaccines.net/newpage114.htm (http://www.webcitation.org/5YKn9dNpn)	Vaccine Safety	No
11	http://www.fda.gov/fdac/features/2001/401_vacc.html (http://www.webcitation.org/5YKnCG307)	Understanding Vaccine Safety: Immunization Remains Our Best ...	Yes
12	www.nap.edu/readingroom/books/vaccine/ (http://www.webcitation.org/5YKnFCzoa)	Vaccine Safety Forum	Yes
13	www.hhs.gov/nvpo/vacsafe.htm (http://www.webcitation.org/5YKnjRzqe)	CDC National Vaccine Program Office: Vaccine Safety	Yes
14	www.who.int/vaccine_safety/ (http://www.webcitation.org/5YKnmfn9c)	WHO | Global Advisory Committee on Vaccine Safety (GACVS)	Yes
15	www.mercola.com/2003/jan/15/vaccine_benefits.htm (WebCite not available)	Vaccine Safety and Benefits Not Scientifically Proven	No
16	www.vaccineinformation.org/safety.asp (http://www.webcitation.org/5YKnzOd7C)	Vaccine Safety	Yes
17	news.bbc.co.uk/1/hi/health/3640898.stm (http://www.webcitation.org/5YKo2JmE8)	BBC NEWS | Health | Study backs safety of MMR vaccine	Yes
18	pediatrics.about.com/cs/immunizations/a/vaccine_safety.htm (http://www.webcitation.org/5YKo5Eb1S)	Understanding Vaccine Safety	Yes
19	www.iom.edu/report.asp?id=25184 (http://www.webcitation.org/5YKoCXBgt)	Vaccine Safety Research, Data Access, and Public Trust – Institute…	Yes
20	www.Michigan.gov/documents/Vaccine-Safety_7192_7.pdf (WebCite not available)	Vaccine Safety	Yes

The study was reviewed and approved by the Institutional Review Boards at Rice University and the Houston Independent School District. Since all but four of the participants were under the age of 18 (median age 17 years), all of the participants and their legal guardians gave written informed consent. The students were financially compensated for their participation in the assessment activity and were debriefed about the purpose of the study at its conclusion.

Participants were not told the purpose of the study, but rather that they were “helping to assess the suitability of assignments for ‘Bioengineering and World Health,’ a new course for high school students.” The students were asked to search for the terms “vaccine safety” and “vaccine danger” using the Google search engine and then to answer a number of questions regarding the accuracy of the health information on the sites. The students were in the same room when the data were collected, but they worked alone on their own computer. We judged the accuracy of the sites on a simple, single dimension: sites that argued that vaccines were inherently dangerous were judged to be inaccurate (not evidenced based), while sites that argued that vaccines were generally beneficial were judged to be accurate (evidenced based). Sponsored links were excluded from the analysis. We then compared that to the students’ assessments based on their answers to the following question: “Do you think that the sites that pop up on the two searches contain accurate health information? Why or why not?” This question forced the students to make a collective assessment of the accuracy of the sites they had just viewed. Table 1 lists the sites that were returned by the Google search for “vaccine danger,” and Table 2 lists the sites returned for “vaccine safety.” In both tables, the list of sites is in the same order as that returned by the search engine.

 At the end of the assessment exercise, the students were asked to write down what they learned from the assignment, and approximately half of the students (n = 17) also filled out a survey that had questions regarding the strength of evidence for seven aspects of vaccinations. These questions required the student to indicate the level of scientific evidence supporting each statement based on the information the students collected. This survey included the following statements: (1) Vaccines have contributed to the eradication of certain diseases; (2) Diseases had already begun to disappear before vaccines were introduced because of better hygiene and sanitation; (3) Vaccines prevent childhood deaths; (4) Vaccines cause autism; (5) Vaccines cause diabetes; (6) Vaccines prevent epidemics; and (7) Vaccines weaken the immune system.

### Methods for the Video Exercise

Because of the surprising (and troubling) results from the search study, these same students were invited to participate in a follow-up study in which a video from the Children’s Hospital of Philadelphia’s Vaccine Education Center entitled “Vaccines: Separating Fact from Fear” was shown [9]. This short, 27-minute film addressed common misconceptions about vaccines and answered each misconception with a fact-based answer. The follow-up study was conducted within 7 days after each participant’s initial visit, and 31 of the original 34 students were able to participate. When the students were asked to participate in this follow-up study, they were simply told that their participation in assessing another module of the course was desired. After watching the video, the students were again asked to write down what they learned from the assignment and to complete the same questionnaire that inquired about the strength of evidence for seven aspects of vaccinations. Simple *t* tests (2-tailed, alpha = .05) were conducted to assess if the responses to the strength-of-evidence questions were different following the video exercise.

## Results

### Results From the Search Exercise

**Figure 1:**
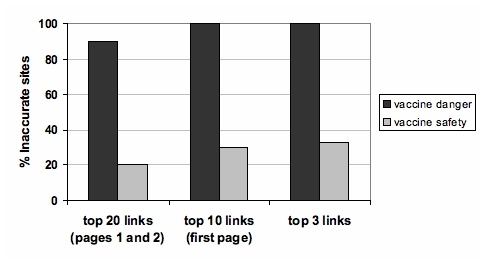
Percentage of inaccurate sites for three different levels of search results

Combining the search results for both search terms (vaccine danger and vaccine safety), it was found that 22 of the 40 links (55%) in the first two pages (40 total search results across both search terms) were inaccurate. Frequently, users restrict their exploration of search results to the first page of results that are returned [10], and this increases the percentage of inaccurate sites to 65% (26 out of 40 links). In a study of how people search for health-related information on the Internet, Eysenbach and Köhler showed that the first three links on a search results page account for approximately 80% of the subsequent click-throughs [11]. Using this measure, 67% (27 out of 40 links) of the sites returned from the Google search were inaccurate. Figure 1 shows the percentage of inaccurate sites for three different levels of search results (2 pages, 1 page, and top 3 links) for each of the search terms used in the study. The percentage of sites found to be inaccurate for the Google search using our simple decision rule is consistent with results reported by Abbott [12]. Clearly, the probability of encountering inaccurate information is very high given the content of the sites that are most likely to be looked at following a search. In fact, if a user searched the term “vaccine danger” only, the first page of search results would have contained no accurate sites.

Student assessment of the accuracy of the returned sites indicated that 20 out of 34 participants (59%) thought that the sites were *accurate* on the whole, while 9 out of 34 participants (26%) thought that the sites were *inaccurate* on the whole. Only 5 students out of the group of 34 (15%) thought that site accuracy was mixed, with some sites being accurate on the whole and others not. These results are consistent with the results of a Pew Research study [13] that found that 52% of those visiting health websites believe that almost all or most of the information is correct.

 A high percentage of the students left the exercise with significant misconceptions about vaccines, based on an analysis of the lessons-learned question they were asked to complete following their search. In this exercise, 18 of 34 participants (53%) provided inaccurate statements about vaccines. Of the 41 verifiable facts about vaccines that were reported by participants in their lessons-learned answers, 24 facts (59%) were *incorrect.* These incorrect facts included statements such as “vaccines can cause diabetes,” “vaccines can cause other diseases later in life,” and “children are diagnosed with autism due to a number of mandatory vaccines.”

### Results From the Video Exercise

**Figure 2:**
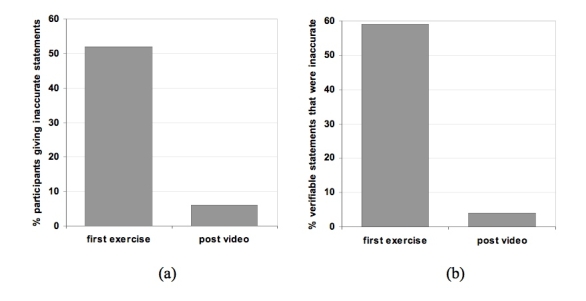
(a) Percentage of students who gave inaccurate statements in the first exercise and after the video; (b) percentage of inaccurate statements given by students in the first exercise and after the video

After completing the second portion of the study, where students watched a film refuting vaccine myths, the majority of students left the exercise with correct information about vaccines, based on their short lessons-learned statement. In this case, 29 of the 31 students (94%) reported accurate information about vaccines. Of the 49 verifiable facts about vaccines that were reported by participants in their lessons-learned answers, only two (4%) were incorrect (Figure 2)*.*

**Figure 3:**
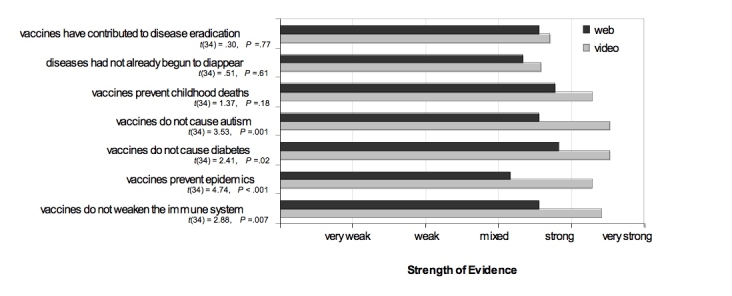
Average response to questions about the strength of evidence for statements about vaccines (dark bars are scores collected after the Web search exercise; light bars are scores collected from the same participants following the video exercise)

Most of the facts reported by the students after the second study centered on the idea that vaccines did not cause other diseases and that while there were certainly risks associated with vaccines, the benefits far outweighed these risks. Following the presentation of the film, students also scored significantly higher (alpha = .05) on most of the questions regarding the strength of evidence for the statements about vaccine facts (Figure 3).

## Discussion

### Principal Results

These results show that even high school students with a science-focused education have a difficult time distinguishing trustworthy medical sites from untrustworthy ones, even though the majority of the information found in these searches is, in fact, inaccurate. Further, the lessons they report learning tend to reflect the most inaccurate information they encountered.

The tasks performed by the students in this exercise are similar to the information- gathering tasks performed by countless consumers of Internet health information every day, and many of these consumers (about 90 million in the United States alone) find medical information hard to understand [14]. Considering the fact that the majority of the information found by these searches on this particularly contentious health topic was *inaccurate* and that over half of the people who visit health websites tend to believe that the majority of the information they find there is *accurate* [13], it quickly becomes evident that there may be significant inaccuracies in the information that people take away from their efforts to gather information on health-related issues.

The problem is of particular concern in an educational setting. It is obviously undesirable to have students engage in Internet-based, information-gathering exercises if they leave the classroom with learnings that are the exact opposite of the intended message. The majority of the students in this exercise initially took away information about vaccines that was substantially incorrect. Their primary misconceptions were that that the risks associated with vaccines outweighed the benefits and that vaccines actually caused, rather than prevented, many diseases. These lessons were reported by the majority of the students even though the intent of the exercise was to teach students about the importance of vaccines in improving health. Given the potentially negative consequences of having students walk away with such erroneous health information, it is important to consider how to best guard against this undesirable outcome.

### Guiding Student Internet Use

The easiest method to insure a specific outcome would be to simply restrict the websites that students are allowed to use during the course of completing an exercise in class to those sites that are deemed reputable and accurate by the instructor. For example, in the exercise described in this paper, the instructors could have directed the students to sites known to be accurate, such as the National Institutes of Health and the World Health Organization, to find information regarding vaccines. While this approach will likely yield the desired immediate result of getting accurate information to the students, it could potentially reinforce the students’ idea that health information on the Web is generally accurate, when in fact the opposite appears to be true. It also fails to train students to think independently about the accuracy of information they find—a skill that is very important in later endeavors involving any information acquisition.

Another potentially simple way to steer students to accurate sites would be by identifying reputable medical sites through the use of “trusted authority” rating systems. These systems work in a fashion similar to the association of a business with the Better Business Bureau or to the ratings of products by an independent organization like Consumers Union. While these systems enjoy widespread adoption in certain domains, the Internet does not currently have a widely accepted trusted authority. Gagliardi and Jadad [15] and Pandolfini and Bonati [16] provide an overview of Internet rating systems. The fundamental flaw with rating systems on the Internet, however, is that there is no central controlling authority. This means that sites are not required to have their content reviewed or rated, and rating systems are not required to be proven valid or impartial. Because the use of rating and review systems is still haphazard, this method of steering students to accurate content is likely insufficient as well.

In the past, health information was often obtained through intermediaries, which were trusted figures like doctors and nurses. The rise of the Internet has given information seekers the chance for greater autonomy through the use of so-called apomediaries [17,18]. Unlike a traditional intermediary, who is a gate-keeper of information, apomediaries help a user find information. The Web itself is, of course, one form of apomediary, but generally the apomediation takes the form of advice given by other users on the Web by way of site recommendations, blogs, or even topic-specific information. This apomediation allows the information seeker to get information from a number of sources and exercise judgments about the credibility of the sources based on the collective preponderance of evidence they have encountered. If the apomediaries are deemed trustworthy in the eyes of the user, then this method can be beneficial, provided that the information supplied by the apomediaries is, in fact, accurate.

Ideally, students and consumers of health information should be trained to critically evaluate the information they find on the Web [19,20]. This can be a complex undertaking [21] because people use a variety of methods to determine the trustworthiness of a site. One method is the slow buildup of trust in a site through extended use [22]. This is a common technique for the selection of trusted news sites [23]. While the trust model may be effective, it is a lengthy process and does not lend itself well to the acquisition of knowledge for which the user desires to access only once. Further, it does not aid in the recognition of reliable sources based on the results of an Internet search.

In a search environment, users must quickly sort through various search results to make a determination of what information they are going to use (ie, information they place provisional trust in) and what information that they are going to discard (ie, information that they have deemed to be untrustworthy). Users employ a number of heuristics to make this initial determination, and, unfortunately, most of the techniques used have little to do with the actual content. One of the most common heuristics is design feature analysis [24], in which users gauge trustworthiness based on the physical design attributes of the site. However, Kunst and colleagues [25] have shown that in the Internet realm there is little correlation between the physical design attributes of a site and the reliability of the information contained on that site. Even if the physical attributes of the site follow presentation guidelines specific to medical information websites [26,27], the correlation remains low [25,28]. Another dimension related to page style is the use of scientific jargon in the presentation of the content. The use of scientific jargon in the presentation of medical information tends to increase the degree to which consumers are persuaded by the material [29]. Since both accurate and inaccurate sites tend to use similar language, a site’s use of medical jargon is probably not helpful in assessing the validity of the site.

Other dimensions of the heuristic analyses include the assessment of the source based on the degree to which the author is viewed as an authority. Name or title recognition is one way users make this assessment. The validity of this technique has not yet been established in the literature, but it is a technique that is commonly employed by users [11,30]. Interestingly, the authority of the source appears to be greatly discounted in the presentation of personal anecdotes. If information has high face validity, users may ascribe more trust to the source than would be warranted upon close inspection of the facts. Some students in this exercise reported that the inclusion of personal stories and testimonials on the websites was highly compelling. Surprisingly, these students also reported that the opposite was true as well: sites that had an abundance of information that was presented in an authoritative, business-like fashion (often in the form of links to peer-reviewed material) were viewed as *less compelling*. It is not known how compelling websites change the trust equation, but data from these participants suggest that anecdotal information carried significant weight since this information was very likely to be reported by the students when they described what lessons they had learned from the exercise. The technique appears to be equally effective for the presentation of accurate information as well. The film used in the follow-up session, while factually accurate and produced by a reputable organization, contained a significant number of stories and anecdotes by parents whose children were protected by vaccinations. The film used a trusted authority (a doctor) to lead the narrative, but his fact-based presentation was always accompanied by an anecdotal story presented by a “real person.” Thus, the establishment of emotional appeal, even for the presentation of fact-based evidence, appears to be of high importance.

### Conclusions

This paper presents a cautionary tale about using the Internet as an instructional tool for controversial medical material. Letting students use the Internet to gain information should be approached with care since students may come away with an incorrect message. While restriction of unstructured Internet activities may be the simplest solution, it does not train the students to use this valuable resource with a critical eye outside the classroom. It is very important to follow up conflicting information, like that commonly found on the Internet, with a solid, unambiguous message that communicates those lessons that the instructor wants the students to take away. This final message delivered to the student should be fact-based, but may need to contain an anecdotal component to counter the strong emotional message that is often delivered by inaccurate sites [31].

Instructors also need to insure that the intended message of the lesson is the one that students have actually retained over the course the instruction. By demonstrating to the students that health information on the Internet is highly variable in its accuracy, and that attributes that commonly influence trust (eg, authority figures, physical design, URL name) may not be good predictors of site accuracy, instructors can help students begin to develop the critical analytical skills necessary to assess the accuracy of Internet information. By presenting both accurate and inaccurate sites for the students to evaluate, the instructor can ensure that not only are the students leaving the classroom with the right information for the specific lesson at hand, but that they also leave a bit more prepared to make these critical evaluations in the real world when they become actual consumers of health information.
